# KCNK5 channels mostly expressed in cochlear outer sulcus cells are indispensable for hearing

**DOI:** 10.1038/ncomms9780

**Published:** 2015-11-09

**Authors:** Yves Cazals, Michelle Bévengut, Sébastien Zanella, Frédéric Brocard, Jacques Barhanin, Christian Gestreau

**Affiliations:** 1 Laboratoire de Neurosciences Intégratives et Adaptatives (UMR7260), Fédération de Recherche 3C (Cerveau, Comportement, Cognition), Aix-Marseille-Université and CNRS, Marseille 13331, France; 2 Centre de Recherche en Neurobiologie et Neurophysiologie de Marseille (UMR7286), Aix-Marseille-Université and CNRS, Marseille 13344, France; 3 Institut de Neurosciences de la Timone (UMR7289), Aix-Marseille Université and CNRS, Marseille 13005, France; 4 Laboratoire de Physio-Médecine Moléculaire (UMR7370), Université de Nice-Sophia Antipolis and CNRS, Nice 06107, France; 5 Laboratories of Excellence, Ion Channel Science and Therapeutics, France

## Abstract

In the cochlea, K^+^ is essential for mechano-electrical transduction. Here, we explore cochlear structure and function in mice lacking K^+^ channels of the two-pore domain family. A profound deafness associated with a decrease in endocochlear potential is found in adult *Kcnk5*^−/−^ mice. Hearing occurs around postnatal day 19 (P19), and completely disappears 2 days later. At P19, *Kcnk5*^−/−^ mice have a normal endolymphatic [K^+^] but a partly lowered endocochlear potential. Using Lac-Z as a gene reporter, KCNK5 is mainly found in outer sulcus Claudius', Boettcher's and root cells. Low levels of expression are also seen in the spiral ganglion, Reissner's membrane and stria vascularis. Essential channels (KCNJ10 and KCNQ1) contributing to K^+^ secretion in stria vascularis have normal expression in *Kcnk5*^−/−^ mice. Thus, KCNK5 channels are indispensable for the maintenance of hearing. Among several plausible mechanisms, we emphasize their role in K^+^ recycling along the outer sulcus lateral route.

## Abstract (web summary)

Potassium is necessary for the mechanical-electrical transduction needed for hearing. Here the authors study mice lacking the potassium channel KCNK5 and show that these channels are mostly expressed in the outer sulcus and are required for hearing, pointing to their essential role in potassium recycling.

## Graphical abstract

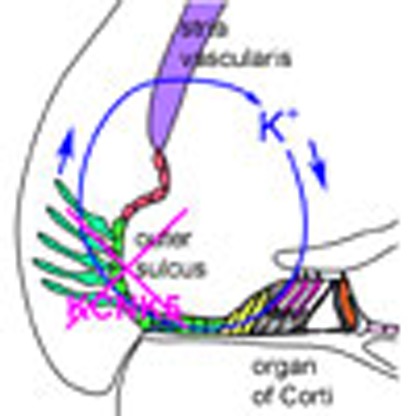

In cochlear hair cells, conversion of sounds into electrical impulses relies on mechanical activation of their apical cationic channels. K^+^ entering across these channels depolarizes the hair cells leading to signal transduction and synaptic transmission at the afferent auditory nerve. K^+^ is then released into extracellular spaces through the hair cell basolateral membranes and recirculated back to the endolymph mostly by its secretion from the stria vascularis. The molecular and cellular basis for the K^+^ recycling mechanism remain quite hypothetical^1^,^2^,^3^,^4^, although it has long been established that K^+^ supply to the stria vascularis originates more from perilymph than blood^5^. Several lines of evidence suggest that K^+^ is recycled through distinct intracellular routes. Indeed, early studies pointed out the role of non-sensory cochlear cells after K^+^ release from outer hair cells; this lateral pathway involves supporting cells (Deiters' and Hensen's cells), outer sulcus cells (Claudius', Boettcher's and root cells), spiral ligament fibrocytes and stria vascularis cell layers^6^. Another route has also been proposed from the inner hair cells to endolymph through a medial pathway involving border and inner sulcus cells, limbal fibrocytes and interdental cells^7^. Pathways contributing to K^+^ cycling may not be limited to these^8^, as K^+^ may also be directly reabsorbed from perilymph by both limbal and spiral ligament fibrocytes, as well as from endolymph through outer sulcus cells although this path seems limited to the cochlear apical turns^6^,^9^,^10^.

Gene mutations that cause deafness highlight the importance of the sophisticated assembly of channels, transporters and gap junctions required for intracochlear K^+^ recycling^2^,^3^. The K^+^ channel KCNQ4 that probably forms the major pathway used by K^+^ to exit the hair cells is located into their basolateral membranes^11^,^12^. *Kcnq4* deletion leads to progressive hair cell degeneration and deafness in humans lasting from early childhood to puberty^11^. Similarly, knockout mice lacking the K^+^–Cl^−^ co-transporter KCC3 (ref. 13) or KCC4 (ref. 14) suffer from hair cell degeneration and deafness. However, *Kcc3* deletion triggers a progressive hearing loss spanning the animal's first year of life^13^, whereas *Kcc4* deletion produces a rapid deafness starting within a couple of weeks after the hearing onset^14^. Another major player that mediates K^+^ transport has been characterized as the inward rectifier KCNJ10 channel expressed in root cells^4^,^15^ and in intermediate cells of the stria vascularis, where this channel is a key element in generating the endocochlear potential (EP)^2^,^16^. Consistent with this, greatly compromised hearing in *Kcnj10* mutant mice is associated with the absence of an EP, and reduced endolymphatic [K^+^] and volume^16^. Gene inactivation for the Na^+^–K^+^–2Cl^−^ co-transporter NKCC1 expressed in outer sulcus, spiral ligament and marginal cells, induces a progressive deafness that starts in juvenile mice^17^ and evolves over several dozens of weeks^18^. Finally, connexins 26 and 30 whose mutations are common causes of genetic deafness in humans are located in the gap junction networks, including the supporting cells, the spiral ligament fibrocytes, and both basal and intermediate cells of the stria vascularis^19^,^20^. This short review supports the concept of K^+^ being transported through networks of cells. However, alternative pathways other than these described above cannot be excluded, and the relative importance of any one of these pathways is still unclear.

Two-pore domain potassium (K2P) channels produce background or leak K^+^ currents over a large range of membrane potential^21^,^22^. In the brain and the kidney, they play major roles in setting membrane potential, pH homeostasis and/or reabsorption of bicarbonate^23^,^24^,^25^,^26^. Interestingly, significant levels for KCNK1, 2, 5, 6 and 17 have been detected from a survey of various inner-ear complementary DNA collections^27^. Indeed, in the inner ear, several members of the K2P channel family have been identified in both vestibular (dark cells and afferent nerve fibres) and cochlear (stria vascularis, spiral ganglion cells and Reissner's membrane) components^28^,^29^. Changes in profiles of K2P channel expression across development suggested various roles for K2P channels in postnatal maturation of the cochlea, K^+^ recycling and/or fluid homeostasis^30^, but their potential role in auditory function has not been examined. Therefore, we analysed the implication of several K2P channels (KCNK3, KCNK5 and KCNK9) in hearing. We found that only *Kcnk5* knockout mice had severe auditory impairment, whereas *Kcnk3* and/or *Kcnk9* gene deletion produced no obvious hearing deficit. Deafness in null *Kcnk5* mice appeared extremely early, within 2 days after the hearing onset. The organ of Corti showed progressive morphological alterations, with early damage of the sensory hair cells and supporting cells in young mice, followed by complete disappearance of epithelial outer sulcus cells above the basilar membrane in the adults, that is Claudius' and Boettcher's cells. Adult, but not juvenile, *Kcnk5*^−/−^ mice also presented a late degeneration of spiral ganglion neurons. These structural changes were associated with an age-dependent decrease in EP. However, the expression of KCNJ10, KCNQ1 and pendrin was not altered. Overall, our results demonstrate an essential role of KCNK5 in the maintenance of hearing. Our data support the hypothesis that KCNK5 in outer sulcus cells play an important role in K^+^ recycling. Other mechanisms mediated by KCNK5 channels including endolymphatic pH-buffering, K^+^ secretion and/or leakage and reabsorption may also contribute to the present findings.

## Results

### Deletion of *Kcnk5* but not *Kcnk3* and/or *9* alters hearing

**Figure 1:**
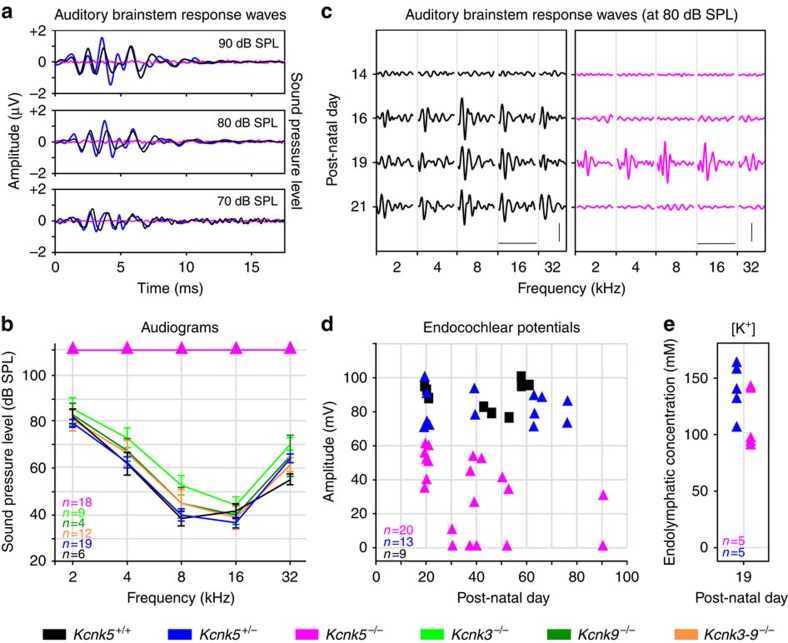
Auditory physiology. (**a**) Superimposed auditory brainstem responses (ABR) waves of *Kcnk5*^*+/+*^ (black, *n*=1), *Kcnk5*^*+/*−^ (blue, *n*=1) and *Kcnk5*^−/−^ (magenta, *n*=1) mice to a 8-kHz tone pip delivered at three sound pressure levels (dB SPL). (**b**) Mean audiogram values (±s.e.m.) obtained from *Kcnk5*^+/+^ (black, *n*=6), *Kcnk5*^+/−^ (blue, *n*=19), *Kcnk5*^−/−^ (magenta, *n*=18), *Kcnk3*^−/−^ (light green, *n*=9), *Kcnk9*^−/−^ (dark green, *n*=4) and *Kcnk3-9*^−/−^ (orange, *n*=12) mice. (**c**) ABR waves recorded from one *Kcnk5*^*+/+*^ (left panel) and one *Kcnk5*^−/−^ (right panel) mice across postnatal days 14–21 as a function of tone-pip frequency (kHz) delivered at 80 dB SPL. Scale bars, 10 ms, 2 μV. (**d**) Individual values of the endocochlear potential recorded from the basal cochlear turn from *Kcnk5*^+/+^ (black, *n*=9), *Kcnk5*^+/−^ (blue, *n*=13) and *Kcnk5*^−/−^ (magenta, *n*=20) mice at different ages from postnatal day 19 to 90. (**e**) Values of endolymphatic K^+^ concentration at the basal turn of the cochlea in *Kcnk5*^+/−^ (blue, *n*=5) and *Kcnk5*^−/−^ (magenta, *n*=5) mice at postnatal day 19.

The role of K2P channels in hearing was first tested in adult mice (P30–P90) by recording far-field auditory brainstem-evoked potentials. Similar patterns of auditory brainstem response (ABR) waves were recorded from all *Kcnk3*^−/−^ (*n*=9), *Kcnk9*^−/−^ (*n*=4), *Kcnk3-9*^−/−^ (*n*=12), *Kcnk5*^*+/+*^ (*n*=6) and *Kcnk5*^*+/*−^ (*n*=19) mice, whereas no signal was detected from the *Kcnk5*^−/−^ (*n*=18) mice. Figure 1a presents ABR waves for the three *Kcnk5* mice types. Audiograms (Fig. 1b), displaying the mean acoustic pressure values of the ABR thresholds for a wide range of frequencies, showed a similar acoustic sensitivity (no statistical difference) in all tested mice but the *Kcnk5*^−/−^ animals, which had no detectable response at any frequency, even for the highest sound pressure level (SPL) tested (110 dB SPL). Furthermore, recordings from the round window performed on three additional *Kcnk5*^−/−^ adult mice confirmed the lack of evoked potential in the 8^th^ nerve for all frequencies and SPLs tested, thus verifying the loss of auditory function. Monitoring the postnatal development through ABR waves was performed between P14 and P21 (Fig. 1c). In both *Kcnk5*^+/+^ (*n*=9) and *Kcnk5*^+/−^ (*n*=8) mice, ABR waves appeared by P15–P16 and were similar between the two groups. A slightly delayed onset of hearing was observed in *Kcnk5*^−/−^ (*n*=6) mice, their ABR waves appearing at P17 (*n*=1) and at P19 (*n*=5). At P19, similar thresholds were measured in *Kcnk5*^+/+^, *Kcnk5*^+/−^ and *Kcnk5*^−/−^ mice. However, ABR waves had disappeared by P21 in all the six tested *Kcnk5*^−/−^ mice.

### Decreased EP in *Kcnk5* ^−/−^ mice

Recordings of the EP were performed in *Kcnk5*^+/+^ (*n*=9), *Kcnk5*^+/−^ (*n*=13) and *Kcnk5*^−/−^ (*n*=20) mice from P19 to P90 (Fig. 1d). At P19, that is, at hearing onset, *Kcnk5*^−/−^ mice had significantly lower EP values (*n*=7, 50.8±9 mV, mean±s.e.m., *P*<0.01) than those measured in both *Kcnk5*^+/−^ (*n*=5, 82.3±14 mV) and *Kcnk5*^+/+^ mice (*n*=5, 92.0±4.6 mV). But at this early age, monitoring the endolymphatic K^+^ concentration in the basal turn (Fig. 1e) showed no significant difference between [K^+^] values in *Kcnk5*^+/−^ (*n*=5, 140.2±22 mM) and *Kcnk5*^−/−^ (*n*=5, 113.2±26 mM) mice. In adult mice (P30–P90), mean EP values were similar in *Kcnk5*^+/+^ (*n*=7, 86.9±10 mV) and *Kcnk5*^+/−^ (*n*=8, 84.1±8 mV) mice, but significantly reduced in *Kcnk5*^−/−^ mice (*n*=13, 22.7±21 mV, *P*<0.001) (Fig. 1d). Linear regression analysis of the EP values measured in *Kcnk5*^−/−^ mice across postnatal development indicated a statistically significant EP decrease with age (*P*=0.036, *R*=0.459).

### KCNK5 does not play an obvious role in vestibular function

Monitoring spontaneous motor behaviours (for example, righting and postural adjustments) did not provide any evidence for a vestibular dysfunction in any of the *Kcnk5*^−/−^ mice tested. In addition, a swim test was conducted on *Kcnk5*^+/+^ (*n*=3), *Kcnk5*^*+/*−^ (*n*=5) and *Kcnk5*^−/−^ (*n*=7) mice of different ages (between P20 and P340) taken from several litters (*n*=8). Not a single sinking episode during the full length of the tests was ever displayed by any one of the tested mice, even by the two oldest (*Kcnk5*^−/−^ of P262 and P340), suggesting no impairment and no delayed degradation of the vestibular function.

### Lack of KCNK5 alters cochlear sensory and neural morphology

**Figure 2:**
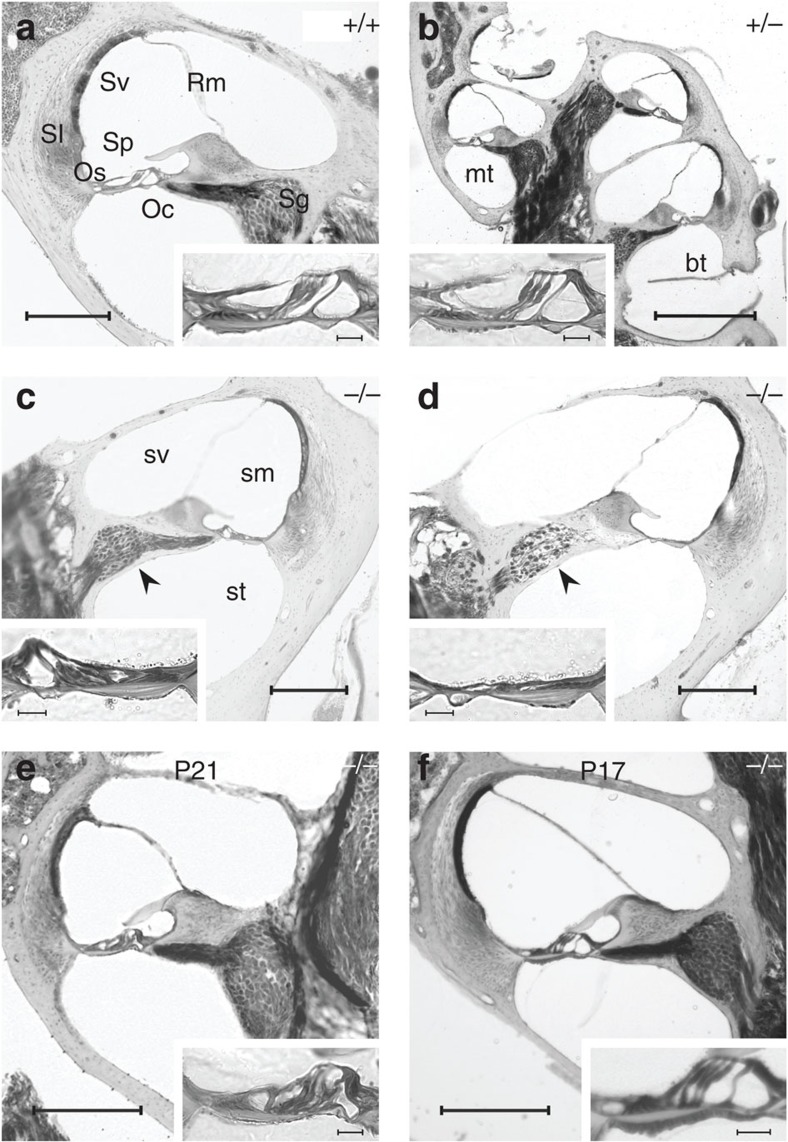
Cochlear structure. The cochlear morphology is illustrated for a whole cochlear section (**b**) and for both basal (bt, **c**,**d**) and medial (mt, **a**,**e**,**f**) cochlear turns; insets show details of the organ of Corti (Oc) and outer sulcus (Os) epithelial cells. Cochlear components are similar in *Kcnk5*^*+/+*^ (**a**) and *Kcnk5*^*+/*−^ (**b**) adult mice. In adult at postnatal day 50 (P50, **c**,**d**) and juvenile (P21, **e**) *Kcnk5*^−/−^ mice, Oc and Os epithelial cells are either altered (**c**,**e**) or absent (**d**); in the adult, the increased neuronal loss in the spiral ganglion (Sg) is correlated with the increased morphological alterations (arrows in **c** and **d**, see text for quantification). In contrast, P17 *Kcnk5*^−/−^ (**f**) mice present normal cochlear components. Additional abbreviations: Reissner's membrane (Rm), spiral ligament (Sl), scala media (sm), spiral prominence (Sp), scala tympani (st), stria vascularis (Sv), scala vestibuli (sv). Scale bars, 200 μm (**a**,**c**–**f**); 500 μm (**b**); 20 μm (insets).

The cochlear gross morphology was similar in *Kcnk5*^+/+^ (*n*=9), *Kcnk5*^+/−^ (*n*=13) and *Kcnk5*^−/−^ (*n*=18) mice. The fine cochlear organization studied in resin sections showed no morphological difference in cochlear coils and scalae (Fig. 2). In adult mice, similar structural and cellular components were found in *Kcnk5*^+/+^ (*n*=6, Fig. 2a) and *Kcnk5*^+/−^ (*n*=5, Fig. 2b) cochleas. In *Kcnk5*^−/−^ cochleas (*n*=7, Fig. 2c,d), both the organ of Corti, the supporting cells and the outer sulcus epithelium showed severe morphological alterations. In adult mice, sensory hair cells and supporting cells had either an abnormal structure (Fig. 2c) or were completely absent with additional disappearance of outer sulcus epithelial cells located above the basilar membrane (Fig. 2d). Similar alterations of the organ of Corti were already present at P21 (*n*=3, Fig. 2e), whereas normal cochlear structures were observed at P17 (*n*=2, Fig. 2f). In addition, losses of spiral ganglion cells could be observed in adults in the basal turns (Fig. 2d), but not in basal or medial turns in juveniles (Fig. 2e). Results from quantification confirmed this qualitative observation. There were significant group differences in the number of spiral ganglion cells per section (*F*=4.217, *P*<0.05), as well as in the packing densities of these cells (*F*=22.774, *P*<0.001). *Post hoc* tests revealed a significant decrease (*P*<0.05 for all comparisons) in the number of spiral ganglion cells per cochlear section in adult (P190–260) *Kcnk5*^−/−^ mice (*n*=9, 49±7), compared with adult *Kcnk5*^+/+^ (*n*=9, 120±14), adult *Kcnk5*^+/−^ (*n*=9, 130±32) and P23 *Kcnk5*^−/−^ mice (*n*=9, 131±15). Similarly, the packing density of cells per section in adult *kcnk5*^−/−^ mice (*n*=9, 4.96±0.38 cells per surface unit) was significantly reduced (*P*<0.001 for all tests) compared with the other groups of mice (*n*=9, 8.74±0.51, *n*=9, 9.21±0.27 and *n*=9, 8.15±0.41 cells per surface unit in adult *Kcnk5*^+/+^, adult *Kcnk5*^+/−^ and P23 *Kcnk5*^−/−^ respectively). No significant difference was found between the three other groups (adult *Kcnk5*^*+/+*^, adult *Kcnk5*^*+/*−^ and P23 *Kcnk5*^−/−^). These observations are in line with the usual time course of spiral ganglion neuron loss occurring after hair cell disappearance^31^. In contrast in all animals, the spiral ligament, the stria vascularis and the Reissner's membrane had normal structure and thickness.

### KCNK5 is mainly expressed in cochlear outer sulcus

**Figure 3:**
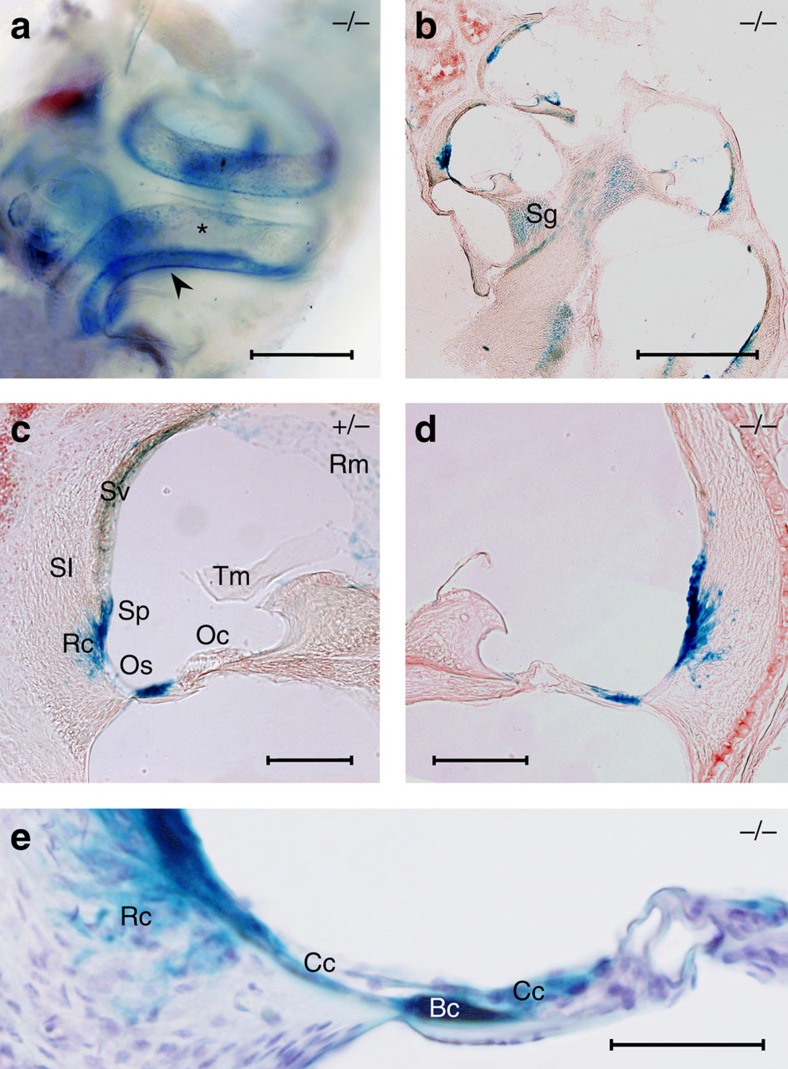
Localization of KCNK5 expression in adult mice. In a *Kcnk5*^−/−^ mouse cochlea *in toto* (**a**) X-gal staining (blue) forms two parallel spiral lines (arrowhead) under the blue spotted-stained (asterisk) stria vascularis (Sv). In sections of *Kcnk5*^−/−^ (**b**,**d**) and *Kcnk5*^*+/*−^ (**c**) mouse cochleas, KCNK5 expression is prominent in outer sulcus (Os) epithelial cells, in the root cells (Rc) of the spiral ligament (Sl) and under the spiral prominence (Sp). A light staining also shows KCNK5 in spiral ganglion (Sg), stria vascularis (Sv) and Reissner's membrane (Rm). When cochleas were reacted together, the X-gal staining in the Os and Rc areas was less pronounced in *Kcnk5*^*+/*−^ (**c**) than in *Kcnk5*^−/−^ (**d**) mice. Details of X-gal staining in a *Kcnk5*^−/−^ (**e**) mouse show KCNK5 present in both Claudius' (Cc) and Boettcher's (Bc) cells. Scale bars: 500 μm (**a**,**b**); 100 μm (**c**,**d**); 50 μm (**e**).

The use of β-galactosidase (β-gal) as a surrogate of KCNK5 expression allowed specific identification of cochlear cells that normally express this K^+^ channel (Fig. 3). In adult mice, X-gal staining of *in toto* cochleas (Fig. 3a) mainly appeared along the cochlear duct as two stripes (arrowhead) below the stria vascularis (asterisk). In cryostat sections of cochleas (Fig. 3b–e), the highest X-gal staining was seen in the outer sulcus epithelial cells and in the root cells under the spiral prominence. Detailed examination of cells located above the basilar membrane shows that both Claudius' and Boettcher's cells express KCNK5. Indeed, the X-gal staining overlaps a small group of cells underlying the Claudius' cells (Fig. 3e) whose location and shape are very similar to those reported by others as Boettcher's cells (see Fig. 4d in ref. 4). A lighter β-gal expression was identified in spiral ganglion neurons, Reissner's membrane and as small spots in few other compartments, including the stria vascularis. Of note, β-gal expression appeared less pronounced under spiral prominence in *Kcnk5*^+/−^ (*n*=4, Fig. 3c) than in *Kcnk5*^−/−^ (*n*=4, Fig. 3d) cochleas.

**Figure 4:**
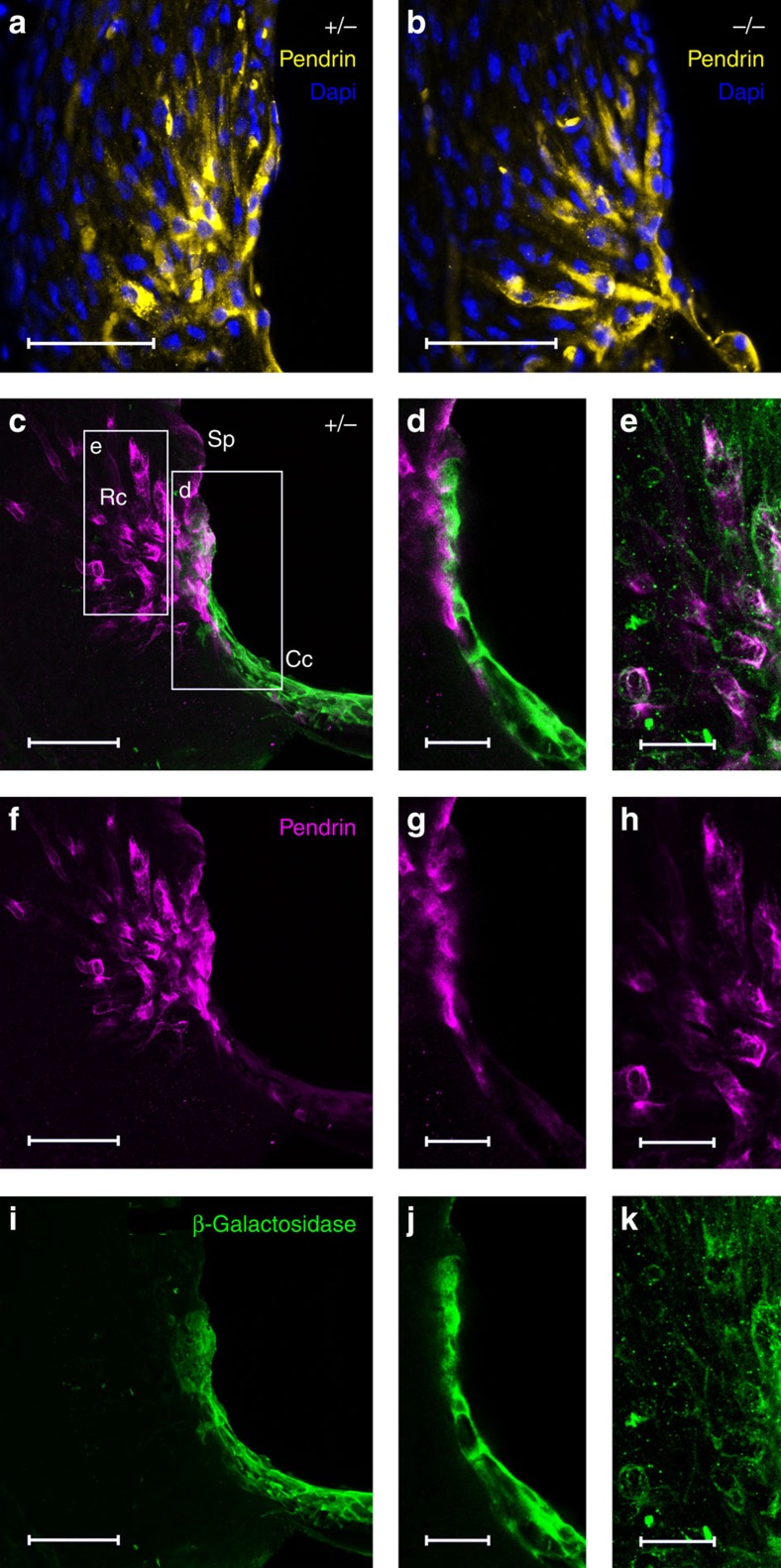
Immunodetection of pendrin and/or β-galactosidase. In cryostat sections from adult cochleas, pendrin (yellow) appears in spiral prominence (Sp) and root cells (Rc) with a similar pattern of expression in *Kcnk5*^*+/*−^ (**a**) and *Kcnk5*^−/−^ (**b**) mice at P45. DAPI (4′,6-diamidino-2-phenylindole dihydrochoride) staining of cell nuclei (blue). In adult *Kcnk5*^*+/*−^mouse cochlea (**c**–**k**), β-galactosidase (green) mainly found in Claudius' cells (Cc, **c**,**d**,**i**,**j**), is also present in Rc (**e**,**k**), while pendrin (magenta) is localized in Rc (**c**–**h**). Merged images (**c**–**e**) show that pendrin and β-galactosidase only colocalize in Rc. Stacks of 23 confocal planes of 1.8 μm each in **c**, **f** and **i**, single confocal planes of 1.8 μm optic thickness in **d**, **g** and **j** and single confocal planes of 0.7 μm optic thickness in **e**, **h** and **k**. Scale bars, 50 μm (**a**–**c**,**f**,**i**); 20 μm (**d**,**e**,**g**,**h**,**j**,**k**).

Since both the outer sulcus root cells and the spiral prominence epithelial cells express pendrin, an anion transporter protein essential for hearing^32^,^33^,^34^, the above results suggested that KCNK5 might be present in these pendrin-expressing cells. Thus, immunohistochemistry was used to detect either pendrin alone or pendrin and β-gal in adult (P45–P60) cochleas of *Kcnk5*^+/+^ (*n*=3), *Kcnk5*^+/−^ (*n*=7) and *Kcnk5*^−/−^ (*n*=7) mice. In whole sections, pendrin was expressed in spiral prominence and in root cells with a similar pattern in all tested mice, including *Kcnk5*^+/−^ and *Kcnk5*^−/−^ animals (Fig. 4a,b). As illustrated in a *Kcnk5*^+/−^ mouse (Fig. 4c–k), confocal imaging confirmed that pendrin is located in the root cells (Fig. 4c–h) and that the highest β-gal staining is found in Claudius' cells (Fig. 4c,d,i,j), some of which being adjacent to the pendrin-positive root cells. A lower level of KCNK5 expression was also observed in the root cells as small puncta or spots of β-gal staining (Fig. 4e,k). Merged images indicated a colocalization of pendrin and KCNK5 in the root cells (Fig. 4e).

### Expression of known K^+^ channels in root cells and stria

**Figure 5:**
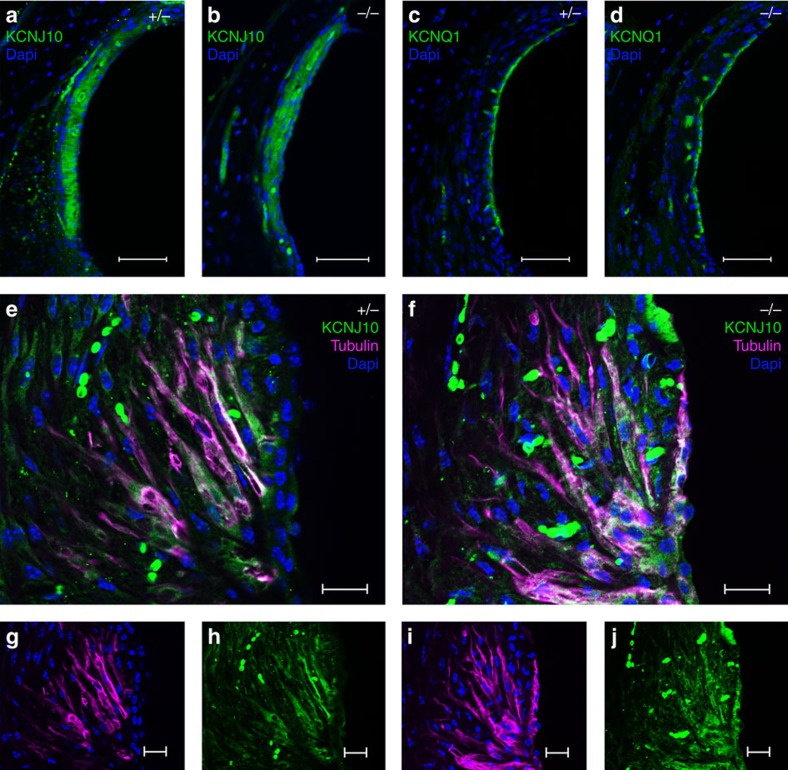
Immunodetection of KCNJ10 and KCNQ1 in adult *Kcnk5*^*+/*−^ and *Kcnk5*^−/−^ mouse cochleas. (**a**–**d**) In both mice groups, KCNJ10 (green) is expressed in the intermediate cells (**a**,**b**), while KCNQ1 (green) is located in the marginal cells (**c**,**d**) of the stria vascularis. Confocal images (stacks of 5 and 2 confocal planes of 2 μm each, respectively, in **e** and **f**) in the area of the root cells (**e**–**j**) revealed similar colocalizations of KCNJ10 (green) and tubulin (magenta) in *Kcnk5*^*+/*−^ (**e**,**g**,**h**) and *Kcnk5*^−/−^ (**f**,**i**,**j**) mice. Cells' nuclei are stained with 4′,6-diamidino-2-phenylindole dihydrochoride (DAPI) (blue, **a**–**j**). Scale bars, 50 μm (**a**–**d**); 20 μm (**e**–**j**).

Additional immunohistochemical experiments were performed to analyse the effect of *Kcnk5* deletion on the expression of KCNJ10 (Kir4.1) and KCNQ1 (Kv7.1), two K^+^ channels playing key roles in K^+^ recycling along the root cells, and EP generation and/or regulation via intermediate and marginal cells of the stria vascularis^35^,^36^,^37^,^38^,^39^,^40^. Qualitative analyses in adult (P46–47) *Kcnk5*^+/−^ (*n*=3) and *Kcnk5*^−/−^ (*n*=3) mice revealed similar patterns of expression of the two K^+^ channels in both groups of mice, with KCNJ10 localized in intermediate cells (Fig. 5a,b) and KCNQ1 expressed in marginal cells (Fig. 5c,d) of the stria vascularis. In the root cell areas of *Kcnk5*^+/−^ (*n*=2, Fig. 5e,g,h) and *Kcnk5*^−/−^ (*n*=2, Fig. 5f,i,j) mice, no obvious difference was found in levels of expression of KCNJ10 and tubulin, a component of the cytoskeleton of the root cell bodies and their processes^15^. Thus, the similar patterns of localization of KCNJ10, KCNQ1 and tubulin suggest no major alteration in their expressions in both stria vascularis and root cells in mice lacking KCNK5.

## Discussion

This study shows that *Kcnk5* knockout mice develop an early and profound deafness within 2 days after hearing onset (that is, P19) associated with an early alteration of sensory hair cells and supporting cells (from P21) followed by a late degeneration of outer sulcus (Os) epithelial cells and of spiral ganglion neurons in adults. In contrast, major K^+^ channels such as KCNJ10 and KCNQ1 involved in K^+^ secretion or recycling have a normal expression in *Kcnk5*^−/−^ mice. These results markedly differ from that obtained after deletion of other genes thought to play a role in K^+^ recycling along the lateral route (*Kcnq4*, *Nkcc1*, *Kcc3* and *Kcc4*), all resulting in progressive deafness over weeks or months^11^,^13^,^14^,^18^,^41^. Thus, our data demonstrate that KCNK5 channels are indispensable for the maintenance of hearing. Since high expression level of KCNK5 was revealed in the Os cells, notably in Claudius' cells, but also in Boettcher's and root cells, the foremost hypothesis is that KCNK5 channels contribute to K^+^ recycling through the lateral epithelial gap junction network^4^,^15^,^42^. Also, our results further suggest that other K^+^ recycling pathways or known K^+^ channels that are expressed in the cochlea seem unable to compensate for KCNK5 loss. In the upper cochlear turn, however, K^+^ recycling may be unaltered since Claudius' cells do not extend over the lower part of the lateral wall under the spiral prominence and since a specific K^+^ reabsorption current has been found there^6^,^9^,^10^. Several preserved mechanisms of K^+^ secretion and/or production of the endolymph, such as those mediated by functional KCNQ1 or KCNE1 subunit channels^43^, are likely responsible for the lack of Reissner's membrane collapse, normal cochlear maturation, adequate endolymphatic K^+^ concentration and at least the partial build-up of the EP that are necessary for hearing onset as observed in P17–P19 *Kcnk5* null mice. In contrast, a few days later, juvenile mice lacking KCNK5 suffered from an early and severe auditory impairment. This is consistent with an indispensable role of KCNK5 channels in sustaining the EP when an increased metabolic demand for K^+^ recycling arises. The lack of significant decrease in [K^+^] measured in *Kcnk5* null mice does not necessarily contradict our hypothesis, since a normal endolymphatic [K^+^] (1) may be achieved via blood supply through the stria vascularis before hearing onset (see introduction), (2) fits with a normal mechano-transduction in very young *Kcnk5*^−/−^ mice and (3) was assessed at the very beginning of audition (at P19) when the EP was high enough to sustain hearing. In *Kcnk5*^−/−^ mice older than P19, we postulate that the lack of proper K^+^ recycling through the Os induces a toxic K^+^ accumulation around sensory hair cells, leading to an early damage of the organ of Corti and to the loss of hearing. The losses of Corti's organ, Os epithelial cells and spiral ganglion neurons observed only in adults were likely secondary to the loss of hearing. It is, however, worthy to note that the normal vestibular function in these mice might be related to a lower requirement for K^+^ recycling to set the vestibular endolymph potential to a few millivolts^8^. Nevertheless, relations between K^+^ concentration in endolymph and EP are complex, as illustrated by a high positive EP associated with a low endolymphatic K^+^ concentration observed in the mouse model of Bartter syndrome^44^. *Kcnk5* knockout mice may represent another interesting model to further investigate these relations. Finally, no detectable cochlear defect or impaired hearing was found in mice lacking KCNK3 and/or KCNK9. Although it has been suggested that KCNK3 could be important in the postnatal development of the cochlea^30^, our data do not support this assumption for any of the K2P channels investigated herein. The slight delay in hearing onset observed in *Kcnk5*^−/−^ mice may suggest that KCNK5 exerts a small effect on cochlear maturation. However, this delay could also be a consequence of the general hypotrophy already shown in these animals^23^.

Interestingly, several membrane ion co-transporters and exchangers may share similar physiological functional profile with KCNK5 channels. Homeostasis of ionic concentrations in the endolymph is achieved by a balance between secretion and reabsorption mechanisms and, both Reissner's membrane and outer sulcus epithelial cells contribute to Na^+^ and K^+^ reabsorption^34^. Since KCNK5 is expressed in these structures, the ionic concentrations in the endolymph may be altered in *Kcnk5*^−/−^ mice. It cannot be ruled out that the lack of KCNK5 alters the integrity of the Reissner's membrane, and thus of the endolymph–perilymph barrier. However the fact that Reissner's membrane presents a normal structure and thickness even in old animals does not favour this hypothesis. In addition to the present demonstration of a crucial role of KCNK5 in hearing, this K^+^ channel contributes to bicarbonate reabsorption by the kidney, and thus participates to the acid–base homeostasis^22^. Mice lacking the K^+^–Cl^−^ co-transporter KCC4 (ref. 14) or the a subunit of the H^+^-ATPase^45^ suffer from renal tubular acidosis associated with hearing loss. Further, lack of pendrin, a Cl^−^/HCO_3_^−^ exchanger involved in endolymphatic pH homeostasis^33^, results in progressive deafness in both humans and mice. Since normal endolymph presents an unusual HCO_3_^−^ concentration, thought to act as a pH buffer^34^, our results showing a strong expression of KCNK5 in the Claudius' cells and its colocalization in pendrin-positive root cells suggest that KCNK5 also contributes to pH-buffering of the endolymph. Therefore, ionic mechanisms involving KCNK5 may be another example of striking similarities occurring between epithelial transport in the inner ear and kidney^35^,^45^. Interestingly, K^+^ currents that are very similar to KCNK5 currents^46^ have been recorded from root cells^15^, but the molecular nature of the K^+^ channel producing these currents remains hypothetical.

It must be noted that KCNK5 channels have different primary structures, tissue localization and pH sensitivity when compared with KCNK3 or KCNK9 channels^21^,^25^,^47^. Indeed, KCNK3 and KCNK9 belong to the TASK subgroup being very sensitive to variations of extracellular pH within the physiological range, while KCNK5 belongs to the TALK subgroup of alkaline-activated K2P channels. KCNK5 expression is highly restricted in both the cochlea (present results) and the brainstem^25^, whereas KCNK3/KCNK9 expression display a more ubiquitous pattern in these organs^30^,^47^. Furthermore, respiratory adaptation to changes in central pH levels critically depends on KCNK5 channels^25^,^26^, but not on KCNK3/KCNK9 ones^24^. In central auditory structures, KCNK3 and KCNK9 are expressed in cochlear spiral ganglion^28^, KCNK3 expression decreases in rat inferior colliculus after cochlear ablation^48^ and KCNK5 was observed in superior olive and inferior colliculus^25^. However, no data are available on their functional roles in these structures. Although our results clearly show that neither KCNK3 nor KCNK9 plays a role in auditory sensitivity, the lack of these channels could be compensated by closely related members of the K2P family, as observed in other physiological functions^21^.

There is presently no human pathology known to be directly related to KCNK5 mutation even though disturbances in cochlear K^+^ recycling are clearly involved in auditory pathology^2^,^3^. There are indications of individual susceptibility to noise-induced hearing loss being linked with gene mutations affecting cochlear K^+^ circulation^43^,^49^. In addition, it has been reported that mice lacking the acid-sensing ion channel 2 were more resistant to noise-induced temporary hearing loss than wild-type (WT) mice^50^. KCNK5 specifically found in outer sulcus epithelial cells in connection with root cells might also be involved in presbycusis, since CBA/CaJ mice, which are affected by early age-related hearing loss, show a concomitant loss of cochlear root cells^51^.

## Methods

### Animals

Juvenile (P14–P23) and adult (from P30 to P340) mice of either sex were used in this study. All experiments were performed on C57BL6/J mouse littermates, bearing or not a mutation for the gene coding for KCNK5, KCNK3, KCNK9 or KCNK3 and KCNK9 (double knockout). Genotyping was performed by a PCR technique on tail DNA using a three primers in the same amplification sample: a forward primer (5′-AGACCAGCCACAACCATACAAT-3′) was common to the WT and knockout products and two reverse primers, one in intron 1 specific for the WT band of 1.2 kb (5′-TAGCAAGGAAGTAGCCAGAGGT-3′) and the other in the introduced exontrap sequence^52^, specific for the knockout band of 0.9 kb (5′-CACTCCAACCTCCGCAAACT-3′). Amplification conditions were 95 °C per 3 min; (95 °C per 20 s; 65 °C per 20 s (−1 °C per cycle); 72 °C per 1 min) 6 × ; (95 °C per 20 s; 60 °C per 20 s; 72 °C per 1 min) 30 ×. The animals had unlimited access to food and water, and were exposed to 12-h light—12-h dark cycles. The experimental procedures were carried out in accordance with French national legislation (JO 87–848, European Communities Council Directive (2010/63/EU,74) and local ethics committee ‘Direction Départementale de la Protection des Populations', with permit numbers 13-47, 13-06 and 13-227 delivered to Y.C., M.B. and C.G., respectively.

### Auditory physiology

Mice were anesthetized with a mixture of ketamine chloride (50 mg kg^−1^) and medetomidin chloride (500 μg kg^−1^) through intraperitoneal injection, and at the end of a recording session, animals were awakened by injection of atipamezol (500 μg kg^−1^, intraperitoneally). All recordings were made in a soundproof chamber.

*Auditory brainstem-evoked potentials*. For auditory brainstem-evoked potentials, mice were placed in front of a loudspeaker (FF1-TDT) and tone pips (with 2 ms linear rise/fall time and no plateau) were delivered at octave frequencies from 2 to 32 kHz at a rhythm of 10 s^−1^. Acoustic levels were measured in dB of SPL with a free-field microphone (Bruel and Kjaer microphone 4191) positioned at the animal's head. ABR waves were recorded via small needle electrodes introduced under the skin at the skull vertex (active electrode), behind one mastoid (reference electrode) and in a neck muscle (ground electrode). Signals were amplified 10^4^ times, filtered between 300 and 3,000 Hz (Grass ICP 511 amplifiers), digitally converted and averaged (10^3^ sweeps) with a Micro1401 Plus system (Cambridge Electronic Devices, UK). Recordings of ABR waves started from an acoustic level of 110 dB SPL and proceeded down in steps of 10 dB. The ABR thresholds for various acoustic frequencies, defined as the lowest intensity (in dB) that generates a clearly identifiable and reproducible response wave, are represented as audiograms.

*Round window recordings*. Round window recordings allow the measure of compound action potentials from the eighth cranial nerve with much larger signal-to-noise ratio than far-field brainstem recordings. They were performed after piercing a small retro-auricular hole in the bulla to gently place a platinum ball electrode into the round window niche.

*EP recordings*. The head of the animal was fixed at frontal bones level to a micromanipulator with screws and acrylic cement. The head was tilted sideways, the pinna was cut, the tympanum and middle ear bones resected and the posterior ridge of the ear canal was gently broken into small pieces with fine tweezers to visualize and access the cochlear first turn. A small hole was pierced with a fine needle through the bone at the level of the stria vascularis pigmented area above the stapedial artery. Then a glass microelectrode (∼10 MΩ) filled with 150 mM KCl solution was slowly inserted through the hole with a micromanipulator while the electric potential was monitored (NeuroLog DC amplifier) and recorded (Micro1401Plus) at least for 2 min.

*K^+^ concentration measurements*. Potassium concentration [K^+^] was measured by means of ion-sensitive microelectrodes^53^. The reference channel was back-filled with 154 mM NaCl, while the ion-sensitive channel was salinized with methyltrichlorosilane in dichloromethane (Fluka/Sigma-Aldrich), tip-filled with the K^+^ ionophore I-cocktail A (Fluka) and back-filled with 100 mM KCl. A differential amplifier subtracted the reference potential from that of the ion-sensitive channel to obtain the pure signal related to changes in the ion concentration. These changes were then calculated using the Nernst equation. The K^+^-sensitive microelectrodes were tested and calibrated using known [K^+^] solutions.

### Vestibular function: swim test

Since auditory and vestibular systems are closely related, we evaluated the role of KCNK5 in vestibular function using a swim test^54^,^55^, on control and *Kcnk5*^−/−^ mice. Mice of either sex were individually lowered in a 20-by-40-cm clear plexiglas aquarium filled with warm water (25–27 °C) to a depth of 20 cm. Control mice displayed a normal swimming behaviour characterized as follows: maintenance of a horizontal body position, nose and tail kept above the water, and swimming towards the side of the cage. The swimming ability was assessed over periods of 60 s by scoring the total duration of sinking episodes, that is, a vertical body position and the nose below the water level.

### Histology of mouse cochlea

*Cochlear samples*. The animals were killed by respiratory arrest under isoflurane overdose. The tympanic bulla was exposed and the otic capsule opened. After removal of the stapes and opening the round window, a small hole was made in the apex of the cochlea, over the helicotrema. Whole cochleas were perfused *in situ* through the round window with either 1% (X-gal staining) or 4% (immunohistological staining) paraformaldehyde in PBS (0.1 M phosphate with 0.15 M NaCl buffered at pH 7.4) or in 2.5% (resin embedding) glutaraldehyde in PBS with 5 mM EGTA, 2 mM MgCl_2_ and 58 mM sucrose. The cochleas were severed from the temporal bone and post-fixed in the same fixative.

*Cochlear structure*. *In toto* cochleas were decalcified in EGTA (500 mM, pH 8) for 48 h, osmicated (2% OsO_4_ in H_2_O), dehydrated in a grade series of alcohols (70, 80, 95 and two 100% baths), cleared in propylene oxide, kept in half propylene oxide and half soft Durcupan resin overnight and then transferred to pure resin. The resin was cured at 60 °C for 48 h. After cooling, each resin block was trimmed so that the embedded cochlea was transversally sectioned in midmodiolar planes. Sections of 10 μm thickness were obtained with a microtome (Reichert-Jung Supercut 2050), serially collected onto glass slides and air dried. They were then covered with pure resin, coverslipped and cured.

*X-gal staining*. The *Kcnk5* mutant mice were generated using an exon-trapping approach, in which the targeting vector contained the *Lac-Z* gene coding for β-galactosidase^55^. Thus KCNK5 expression was localized by staining for β-galactosidase enzyme activity using the X-gal substrate (5-bromo-4-chloro-3-indolyl-β-d-galactopyranoside). *In toto*, 1% paraformaldehyde-fixed whole cochleas were washed in PBS, immersed in staining buffer (PBS, 5 mM EGTA, 2 mM MgCl_2_, 0.01% Na-desoxycholate and 0.02% Nonidet P-40) for 1 h at 37 °C and left overnight at 37 °C after the addition of 0.245 mM X-gal solution (from a stock solution of 5% X-gal in *N*,*N* dimethylformamide), 0.1 mM potassium ferricyanide and 0.1 mM potassium ferrocyanide. After decalcification, cochleas were immersed and positioned in Tissue-Tek OCT compound before freezing (−20 °C). Cryostat sections of 40 μm thickness made in the modiolar cochlear plane were serially collected onto gelatin-chromalun-coated slides. Then, the sections were air dried, hydrated in PBS and counterstained with haematoxylin. Whole cochleas and sections were dehydrated in a grade series of alcohols, cleared in xylene, mounted in DPX mountant for histology (Fluka) and photographed.

*Immunohistochemistry*. Air-dried cryostat sections (see above) from 4% paraformaldehyde-fixed whole cochleas were rehydrated in PBS, kept in a blocking solution (0.1% Triton-X 100 with 1% bovine serum albumin and 1% normal goat serum in PBS) for 30 min at room temperature and then incubated in primary antibody in the blocking solution, overnight at 4 °C—the rabbit monoclonal anti-β-galactosidase (cat. # 55976, Cappel, MP Biomedicals, Solon, OH) was used at 1:500; the rabbit polyclonal anti-pendrin (generous gift from Dr R. Chambrey; an antibody initially characterized by Royaux *et al*.^33^,^56^,^57^) at 1:500; the rabbit polyclonal anti-KCNJ10 (cat. # APC-035, Alomone Lab Ltd, Jerusalem, Israel) at 1:400 or the rabbit monoclonal anti-KCNJ10 (cat. # ab80959, Abcam, Cambridge, UK) at 1:100; the polyclonal rabbit anti-KCNQ1 (cat. # APC-022, Alomone Lab Ltd) at 1:100; and the mouse monoclonal anti-acetylated tubulin (cat. # T7451, Sigma-Aldrich) at 1:1,000. Following several PBS washes, sections were incubated in secondary antibody in 1% normal serum in PBS, in the dark for 2 h at room temperature—the goat anti-rabbit immunoglobulin-G (H+L) Alexa Fluor 488-conjugated (cat.# 111-545-144, Jackson ImmunoResearch, West Grove, PA) was diluted 1:800 and the goat anti-rabbit or anti-mouse immunoglobulin-G (H+L) Rhodamine Red X-conjugated (cat. # 11-295-144 and 115-295-166, Jackson ImmunoResearch), diluted 1:800. After several washes, slides were coverslipped and mounted using Faramount (Dako, Glostrup, Denmark). For confocal microscopy, the sections were counterstained with the blue-fluorescent 4′,6-diamidino-2-phenylindole dihydrochoride for nucleic acid stain (D 9542, Sigma-Aldrich) used at 0.1 μg ml^−1^ before mounting.

### Analysis of spiral ganglion cell loss

High-magnification microphotographs were obtained from resin-embedded cochleas' sections and used for semi-automatic quantification of spiral ganglionic cells. First, all spiral ganglionic cells with clearly visible cell somata were manually plotted, and the spiral ganglionic boundaries were also delimited (Photoshop). Then, the number of cells per section, the surface of tissue (in a.u.) and the relative packing density of cells per section, estimated as the ratio between cell number and surface, were calculated (ImageJ, Wayne Rasband, National Institutes of Health, USA), using three sections from each animal. Mean numbers were then determined in adult *Kcnk5*^+/+^ (*n*=3), adult *Kcnk5*^+/−^ (*n*=3), P23 *Kcnk5*^−/−^ (*n*=3) and adult *Kcnk5*^−/−^ (*n*=3) groups of mice.

### Images and figures

Observations and photographs were made with either an Olympus BX50 or a Zeiss ApoTome (1D-SIM) AxioObserver ZI microscope equipped with both epifluorescence and a high-resolution digital camera either an Olympus DP50 (Olympus Optical, Germany) or a Zeiss AxioCam MRm (Carl Zeiss MicroImaging, Germany), or with a Laser Scanning Confocal Microscope (LSM510; Carl Zeiss MicroImaging). The figures were prepared with Photoshop software (Adobe Systems Inc.) and both brightness and contrast of the images were adjusted.

### Statistics

Data were expressed as mean values±s.e.m. from *n* observations and compared using either a paired *t*-test (two groups) or an analysis of variance (one way) followed by Dunnett's test correction for multiple comparisons. A linear regression analysis was used to test the age effect on EP values recorded in *Kcnk5*^−/−^ mice. Significance level was taken as *P*≤0.05. All computations were performed using SigmaStat software.

## Additional information

**How to cite this article:** Cazals, Y. *et al*. KCNK5 channels mostly expressed in cochlear outer sulcus cells are indispensable for hearing. *Nat. Commun.* 6:8780 doi: 10.1038/ncomms9780 (2015).
